# A new species of rain-pool frog (Dicroglossidae: *Fejervarya*) from western Thailand

**DOI:** 10.24272/j.issn.2095-8137.2017.043

**Published:** 2017-09-18

**Authors:** Chatmongkon Suwannapoom, Zhi-Yong Yuan, Ke Jiang, Fang Yan, Wei Gao, Jing Che

**Affiliations:** ^1^Division of Fishery, School of Agriculture and Natural Resources, University of Phayao, Phayao 56000, Thailand; ^2^Kunming Institute of Zoology, Chinese Academy of Sciences, Kunming Yunnan 650223, China; ^3^College of Forestry, Southwest Forestry University, Kunming Yunnan 650224, China; ^4^Southeast Asia Biodiversity Research Institute, Chinese Academy of Sciences, Yezin Nay Pyi Taw 05282, Myanmar

**Keywords:** *Fejervarya muangkanensis***sp. nov**., Kanchanaburi, Thailand

## Abstract

We describe a new species, *Fejervarya muangkanensis* **sp. nov**., based on a series of specimens collected from Ban Tha Khanun, Thong Pha Phum District, Kanchanaburi Province, Thailand. The new species is easily distinguished from its congeners by morphological and molecular data, and can be diagnosed by the following characters: (1) small size (adult male snout-vent length (SVL) 33.5 mm; female SVL 40.0-40.9 mm); (2) tympanum small, discernible but unclear; (3) poorly developed toe webbing; (4) no lateral line system in adults; (5) characteristic "Fejervaryan" lines present in females; and (6) femoral glands absent. Molecular phylogenetic analysis of mitochondrial 16S rRNA further supports it as a distinct lineage and distinguishes it from its congeners for which sequences are available.

## INTRODUCTION

The genus *Fejervarya* (Bolkay, 1915) (family Dicroglossidae Anderson, 1871) currently contains 41 species (Frost, 2017) and two reciprocally monophyletic species groups (Dinesh et al., 2015) comprising the: (1) South Asian group and (2) East and Southeast Asian group. Thailand has eight species (Frost, 2017), including *Fejervarya* *chiangmaiensis* (Suwannapoom et al., 2016), *Fejervarya andamanensis* (Stoliczka, 1870), *Fejervarya* *cancrivora* (Gravenhorst, 1829), *Fejervarya limnocharis* (Gravenhorst, 1829), *Fejervarya moodiei* (Taylor, 1920), *Fejervarya multistriata* (Hallowell, 1861), *Fejervarya orissaensis* (Dutta, 1997), and *Fejervarya triora* (Stuart et al., 2006). Except for *F*. *andamanensis*, which belongs to the South Asian group, all other Thai species are assigned to the East and Southeast Asian group (Dinesh et al., 2015; Suwannapoom et al., 2016).

Recent morphological and genetic comparisons have revealed several new species of Thai anurans, including one new species of *Fejervarya* from northern Thailand (Suwannapoom et al., 2016). During herpetological surveys in 2013 in the Kanchanaburi Province of Thailand, we found a morphologically distinct population of *Fejervarya*. We compared the morphology of this species with its congeners as well as levels of genetic divergence with species having comparable data in GenBank. These analyses supported the recognition of a new species.

## MATERIALS AND METHODS

### Sampling

**Figure 1:**
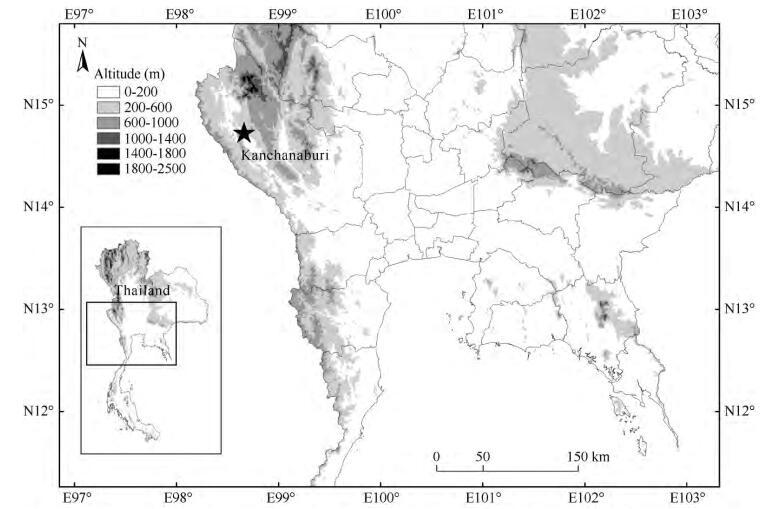
Distribution of *Fejervarya muangkanensis* sp. nov. in western Thailand: Ban Tha Khanun, Thong Pha Phum, Kanchanaburi Province (star: type locality)

Five individuals (KIZ 024627, KIZ 024675-78) were captured during fieldwork in the village of Tha Khanun, Thong Pha Phum District, Kanchanaburi Province, Thailand (Figure 1), from June to September 2013. After euthanization using a chlorobutanol solution, muscle and liver tissues were taken from the frogs and preserved in 95% ethanol for genetic analysis. Specimens were later fixed in 10% buffered formalin and then transferred to 70% ethanol. All specimens were deposited at the Kunming Institute of Zoology (KIZ), Chinese Academy of Sciences (CAS).

### Molecular analysis

Total genomic DNA was extracted from a tissue sample of specimen KIZ 024627 using the standard phenol-chloroform protocol (Sambrook et al., 1989). A fragment of 16S rRNA was amplified for one individual using the primers 16Sar (5′-CGCCTGTTTAYCAAAAACAT-3′) and 16Sbr (5′-CCGGTYTG AACTCAGATCAYGT-3′) from Kocher et al. (1989). Amplification involved an initial cycle of denaturation at 95 ℃ for 5 min, and 35 subsequent cycles of 95 ℃ for 1 min; the annealing temperature was 55 ℃ for 1 min and 72 ℃ for 1 min, followed by a final extension step of 72 ℃ for 7 min. The resulting PCR products were directly cycle-sequenced with the same primers as those used for PCR. Sequence analysis was performed on an ABI PRISM^®^ 3730*xl* DNA Analyzer (Applied Biosystems, UK) at KIZ, CAS.

### Phylogenetic analysis

New sequences were examined for signal quality and confirmed for completeness using DNASTAR 5.0. Nineteen sequences were downloaded from GenBank for analysis (Table 1). *Occidozyga lima* was chosen as an outgroup taxon (Pyron & Wiens, 2011). After trimming the ends, sequences were aligned with gaps using MUSCLE (Edgar, 2004) with default settings. Genetic distances among the taxa were calculated using the *p*-distance model in MEGA 6.0 (Tamura et al., 2013). Phylogenetic reconstructions were executed using Bayesian inference (BI) and maximum likelihood (ML). The best-fit model of DNA sequence evolution was chosen using MrModeltest v2.3 (Nylander, 2004) under the Akaike information criterion. The GTR+I+G model was selected as the best model. A Bayesian tree was generated using MrBayes 3.1.2 (Ronquist & Huelsenbeck, 2003). For BI analyses, two independent searches with random starting trees were run for 5 million generations while sampling over 1 000 generations and compared using four Markov Chain Monte Carlo (MCMC) chains (temp=0.2). Convergence was assessed by plotting the log-likelihood scores in Tracer v.1.5 (Rambaut et al., 2013), and data from the ﬁrst 25% were discarded as burn-in before building a consensus tree. Maximum likelihood analyses were performed using RAxML 7.0.4 (Stamatakis et al., 2008). The same model of nucleotide substitution as for the BI analyses was used for ML tree-searching and nodal stability was estimated with 1 000 bootstrap pseudoreplicates.

**Table 1:** Specimens corresponding to genetic samples included in the phylogenetic analyses

Species	Specimen voucher No.	Locality	GenBank accession No.
*Fejervarya cancrivora*	—	Indonesia: Central Java; Banyumas	AB444690
*Fejervarya caperata*	—	India: Mudigere	AB488894
*Fejervarya* cf. *brevipalmata*	030607-01	India: Western Ghats, Madikeri	AB167946
*Fejervarya* cf. *nilagirica*	—	India: Western Ghats; Kudremukh	AB167950
*Fejervarya* cf. *syhadrensis*	—	India: Kurnool	AB488893
*Fejervarya chiangmaiensis*	KIZ 024057	Thailand: Chiang Mai; Omkoi	KX834135
*Fejervarya granosa*	—	India: Mudigere	AB488895
*Fejervarya greenii*	—	Sri Lanka: Hakgala	AB488891
*Fejervarya keralensis*	WⅡ:3263	India	JX573181
*Fejervarya kirtisinghei*	MNHN 2000.620	Sri Lanka: Laggala	AY014380
*Fejervarya kudremukhensis*	—	India: Kudremukh	AB488898
*Fejervarya limnocharis*	—	Indonesia: Java	AB277302
*Fejervarya mudduraja*	—	India: Madikeri	AB488896
*Fejervarya pierrei*	—	Nepal: Chitwan	AB488888
*Fejervarya rufescens*	030526-03	India: Western Ghats; Mangalore	AB167945
*Fejervarya sahyadris*	RBRL 050714-02	India: Aralam	AB530605
*Fejervarya muangkanensis* **sp. nov.**	KIZ 024627	*Thailand:* Kanchanaburi; Thong Pha Phum	MF166918
*Fejervarya syhadrensis*	—	Sri Lanka	AY141843
*Fejervarya triora*	—	Thailand: Ubon Ratchathani	AB488883
*Occidozyga lima*	—	Malaysia: Kuala Lumpur	AB488903
"—": no available museum Cat. No.			

### Morphological analysis

Measurements from four adult males and one female were made with digital calipers to the nearest 0.1 mm. Eighteen morphometric characters of post-metamorphic individuals were recorded in accordance with Matsui (1984) and included: SVL: snout-vent length; HL: head length; S-NL: snout-nostril length; N-EL: nostril-eye length; SL: snout length; EHD: eye horizontal diameter; T-ED: tympanum-eye distance; HW: head width; IND: internarial distance; IOD: interorbital distance; UEW: upper eyelid width; FLL: forelimb length; LAL: lower arm length; FFL: first finger length; HLL: hindlimb length; TL: tibia length; FL: foot length; and IMTL: inner metatarsal tubercle length. Additionally, we also measured finger lengths (Ⅰ-Ⅳ FL) and toe lengths (Ⅰ-Ⅴ TOEL). The toe-webbing formula followed Savage (1975).

## RESULTS

### Phylogenetic analyses

**Figure 2:**
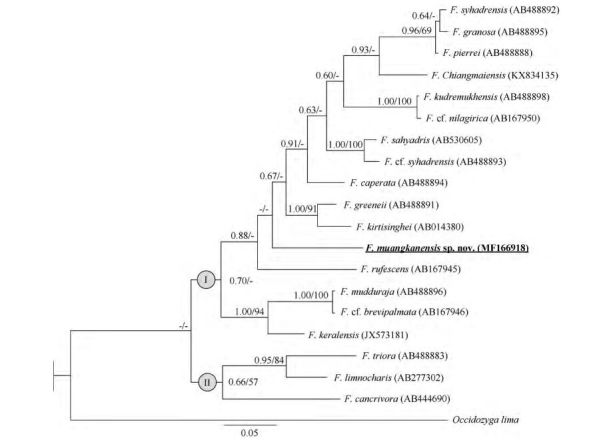
Bayesian inference tree derived from partial fragments of 16S rRNA genes

The unique *de novo* sequence was deposited in GenBank under accession No. MF166918 (Table 1). A total of 721 base pairs (bp) of 16S rRNA data were generated, among which 549 positions were potentially parsimony-informative. Similar topologies were produced by ML and BI analyses. Major clades Ⅰ and Ⅱ were identified within *Fejervarya* (Figure 2), which corresponded to the groups of *Fejervarya* identified by Dinesh et al. (2015). Clade Ⅰ contained the new species plus *F. chiangmaiensis*, *F.* *syhadrensis*, *F.* *granosa*, *F.* *pierrei*, *F.* *kudremukhensis*, *F.* cf. *nilagirica*, *F.* cf. *syhadrensis*, *F.* *sahyadris*, *F.* *caperata*, *F.* *greenii*, *F.* *kirtisinghei*, *F.* *rufescens*, *F.* cf. *brevipalmata*, *F.* *mudduraja*, and *F. keralensis*. Clade Ⅱ consisted of Thai and Indonesian groups, and included *F. triora*, *F. limnocharis*, and *F. cancrivora*. The new population from Thong Pha Phum formed a distinct lineage. However, the relationships among the new population and other species within clade Ⅰ were not resolved. The *p*-distances for 16s rRNA between the new population and other congeners ranged from 8.8% (*F. caperata*) to 13.8% (*F. keralensis*) (Table 2). These results revealed a substantial genetic divergence between the specimens from Thong Pha Phum and the other species, suggesting that this population represents an undescribed species. Furthermore, distinct morphological differences were also found. For example, the "Fejervaryan" lines on legs characteristic, present in females, occurred in the Thong Pha Phum population. Considering its independent evolutionary history, level of genetic divergence, and distinct morphological characters, a new species is described.

**Table 2:** Uncorrected pairwise *p*-distances of 16S rRNA (mtDNA) gene sequences among *Fejervarya* species groups in this study

	A	B	C	D	E	F	G	H	I	J	K	L	M	N	O	P
*F. chiangmaiensis* (A)																
***F. muangkanensis* sp. nov.** (B)	0.115															
*F. syhadrensis* (C)	0.060	0.095														
*F. granosa* (D)	0.057	0.102	0.006													
*F. pierrei* (E)	0.053	0.098	0.006	0.004												
*F. kudremukhensis* (F)	0.086	0.102	0.088	0.091	0.086											
*F. nilagirica* (G)	0.086	0.102	0.088	0.091	0.086	0.000										
*F. syhadrensis* (H)	0.086	0.110	0.084	0.086	0.081	0.086	0.086									
*F. sahyadris* (I)	0.086	0.100	0.086	0.089	0.084	0.083	0.083	0.011								
*F. caperata* (J)	0.086	0.088	0.083	0.086	0.086	0.079	0.079	0.060	0.060							
*F. greenii* (K)	0.093	0.105	0.099	0.101	0.101	0.098	0.098	0.084	0.086	0.072						
*F. kirtisinghei* (L)	0.094	0.108	0.096	0.099	0.099	0.098	0.098	0.081	0.086	0.072	0.042					
*F. rufescens* (M)	0.115	0.110	0.123	0.130	0.125	0.113	0.113	0.118	0.110	0.103	0.098	0.110				
*F. brevipalmata* (N)	0.122	0.135	0.125	0.117	0.122	0.130	0.130	0.125	0.125	0.115	0.110	0.118	0.130			
*F. mudduraja* (O)	0.122	0.135	0.125	0.117	0.122	0.130	0.130	0.125	0.125	0.115	0.110	0.118	0.130	0.000		
*F. keralensis* (P)	0.120	0.138	0.123	0.115	0.120	0.133	0.133	0.121	0.121	0.105	0.110	0.118	0.135	0.051	0.051	

### Species description

**Figure 3:**
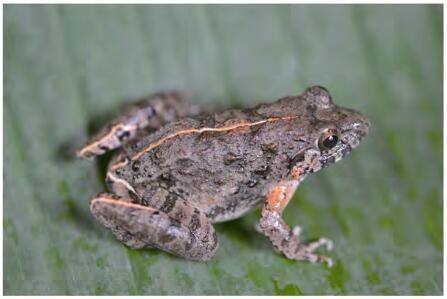
Dorsolateral view of female paratype KIZ 024678 of *Fejervarya muangkanensis* sp. nov. in life (photo by Chatmongkon Suwannapoom)

**Figure 4:**
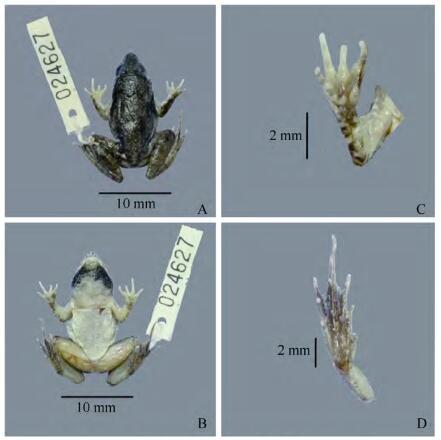
Preserved holotype of *Fejervarya muangkanensis* sp. nov. (KIZ 024627) (photos by Chatmongkon Suwannapoom)

**Figure 5:**
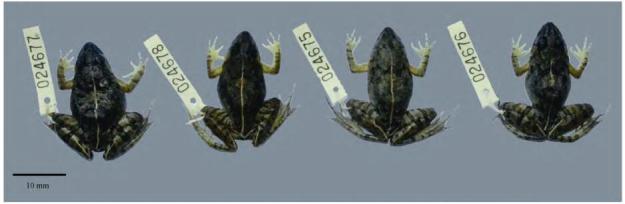
Paratype series of *Fejervarya muangkanensis* sp. nov. (four females) (photo by Chatmongkon Suwannapoom)

***Fejervarya muangkanensis* sp. nov. (Figure 3-5)**

**Holotype:** Adult male (KIZ 024627) from Ban Tha Khanun, Thong Pha Phum, Kanchanaburi Province, Thailand (N15°11'52.73" , E98°19'29.71" ; 712 m a.s.l.), collected by Chatmongkon Suwannapoom at night on 7 August, 2013.

**Paratypes:** Four females (KIZ 024675-78), collected by Chatmongkon Suwannapoom, Jing Che, Fang Yan, and Wei Gao at the same locality as the holotype.

**Diagnosis:** The new species is assigned to *Fejervarya* based on its position in the matrilineal genealogy (Figure 2) and morphological characters of the genus, including slightly pointed snout; comparatively poorly developed foot webbing; lateral line system in adult absent; femoral glands absent; tympanum comparatively small; and tibia length slightly more than half of SVL. The new species differs from its congeners by the following combination of characters (Table 3): (1) medium adult size: male SVL 33.5 mm (*n*=1) and female SVL 40.0-40.9 mm (*n*=4); (2) head longer than wide; (3) tympanum small, discernible but unclear; (4) relative finger length Ⅱ < Ⅳ < Ⅰ < Ⅲ; (5) poorly developed toe webbing; webbing formula: **Ⅰ** 1-2 **Ⅱ** 1-2 ½ **Ⅲ** 2-3 **Ⅳ** 3-1 **Ⅴ**; (6) orange vertebral line present medially, running from under eyes to vent in females (Figure 5); (7) paired dark vocal sacs present in males (Figure 4); (8) tubercles on dorsal and lateral head and body, and body flanks; and (9) posterior part of dorsum with distinct, round glandular warts, continuing on dorsal surface of legs and arms.

**Table 3:** Morphological measurements (mm) of *Fejervarya muangkanensis* sp. nov.

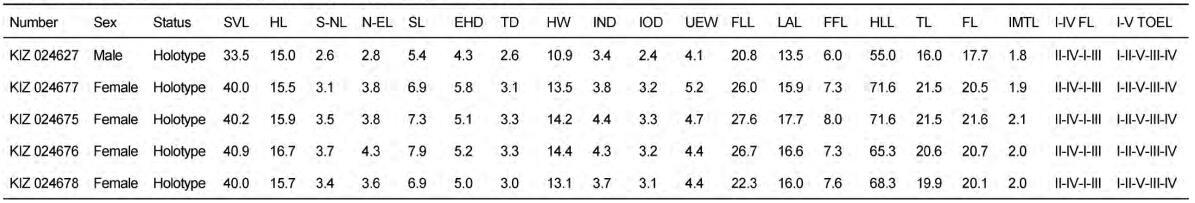

**Description of holotype**: Head size moderate, longer than wide (HW/HL=0.7), convex (Figure 4A). Snout more or less pointed as seen from above; snout length longer than horizontal diameter of eye (SL/EHD=1.3) and interorbital distance (SL/IOD=2.3). Interorbital space slightly convex, much narrower than upper eyelid width (IOD/UEW=0.6) and internarial distance (IOD/IND=0.7). Nostrils rounded, with a distinct flap of skin laterally; nostrils slightly closer to snout than to eye (S-NL= 2.6 mm; N-EL=2.8 mm). Eyes relatively small, protuberant, pupil horizontal.

Forearm short (LAL=13.46 mm), rather strong, 64.8% of forelimb length (FLL=20.8 mm). Fingers short, thin; dermal fringe absent; webbing absent; finger tips bluntly rounded and not enlarged to disks (Figure 4C). Relative finger lengths: Ⅱ < Ⅳ < Ⅰ < Ⅲ.

Hindlimbs relatively long (HLL=55.0 mm), about 1.6 times SVL (33.5 mm). Tibia (TL=16.0 mm) slightly shorter than femur and subequal to foot length (FL=17.7 mm). Toes long and thin, toe tips blunt, slightly rounded, not enlarged to disks (Figure 4D). Relative toe lengths: Ⅰ < Ⅱ < Ⅴ < Ⅲ < Ⅳ. Subarticular tubercles prominent, elongated, and oval-shaped. Inner metatarsal tubercle prominent, long, and slightly compressed laterally (IMTL=1.8 mm). Foot web feeble; webbing formula: **Ⅰ** 1-2 **Ⅱ** 1-2 ½ **Ⅲ** 2-3 **Ⅳ** 3-1 **Ⅴ** (Figure 4C, D).

Snout smooth, with rare indistinct dermal tubercles; nares with low dermal flaps; small tubercles on upper eyelid. Dorsal and lateral surfaces of head and body, including body flanks, shagreened; posterior part of dorsum with distinct, round glandular warts, continuing on dorsal surfaces of legs and arms; small tubercles on the anterodorsal part of thigh, cloacal region, dorsal surface of tibia, and tarsus; lateral sides of body, ventral surfaces of body, and limbs smooth. Dorsal skin showing small, rare, and longitudinal dermal ridges arranged in series.

**Coloration of holotype in life:** Male dorsal ground color varies from brown to dark green, and transverse black bands are present on the dorsal surface of the thigh, tibia, and tarsus region (Figure 3). In females, the mid-dorsal stripe is orange, and bands run from the anterior side between the eyes to the vent and from the posterior side of the thigh to the tarsus; forearm has prominent orange spots, nearly touching the subtympanic orange streak.

**Coloration of holotype in preservative**: Dorsum grayish brown with many large black spots (Figure 4). Thin, cream-colored mid-dorsal stripe runs from between the eyes to the vent and from the posterior side of the thigh to the tarsus (females). Lateral side with many small black dots; ventral side immaculate, except for white with black bands across the throat. Transverse black bands on upper surface of the thigh, tibia, and tarsus to outer edge of the foot.

**Etymology:** The specific epithet muangkanensis is derived from the common name of the Kanchanaburi province, Thailand.

**Suggested common names:** We suggest the following common names: Kanchanaburi Rain-Pool Frog (English).

**Figure 6:**
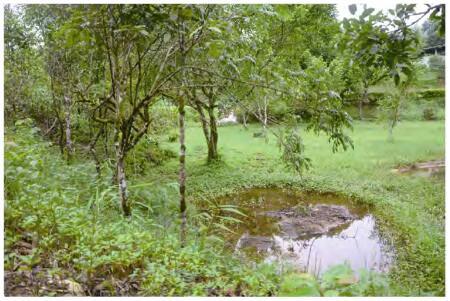
Habitat at the type locality of *Fejervarya muangkanensis* sp. nov., Thong Pha Phum, Kanchanaburi Province, Thailand (photos by Chatmongkon Suwannapoom)

**Ecology:** The species is found in small swamps in secondary forests at elevations between 700-900 m a.s.l. Advertisement calls of the males can be heard in small ponds from July to September in Thong Pha Phum, Kanchanaburi Province. Calling males are usually observed within or beside the swamp (Figure 6).

**Distribution:** This species is currently known only from Thong Pha Phum, Kanchanaburi Province, western Thailand (Figure 1).

**Comparisons:** *F*. *muangkanensis* **sp. nov.** can be distinguished from *F*. *sahyadris* (Western Ghats, India; Ohler et al., 2009) and *F.* *chilapata* (West Bengal, India; Ohler et al., 2009) by differences in body size (male *F. muangkanensis*, *n*=1, SVL=33.5 mm; male *F.* *sahyadris*, *n*=10, mean SVL=18.4 mm; and male *F.* *chilapata*, *n*=8, mean SVL=20.0 mm).

*F*. *muangkanensis* **sp. nov.** can be differentiated from large and medium-sized members of *Fejervarya* (species in the Asian group) by external morphology, coloration, and molecular characteristics. The body size of *F.* *muangkanensis* **sp. nov.** males (SVL=33.5 mm) is larger than that of *F. chiangmaiensis* (SVL=26.3-29.1 mm; Suwannapoom et al., 2016) and *F. granosa* males (SVL=29.1 mm; Kuramoto et al., 2007).

The new species can be distinguished from *F. pierrei* by relative finger lengths, with the second finger being shorter than the fourth finger (Ⅱ < Ⅳ < Ⅰ < Ⅲ vs. Ⅱ=Ⅳ < Ⅰ < Ⅲ; Howlader, 2011). *F*. *muangkanensis* **sp. nov.** can be differentiated from *F. syhadrensis* by its head width being less than head length (HW/HL=0.7; Kuramoto et al., 2007) and by relative finger lengths (Ⅱ < Ⅳ < Ⅰ < Ⅲ vs. Ⅰ=Ⅱ < Ⅳ < Ⅲ; Howlader, 2011).

Although comparative data are limited, the SVL of *F*. *muangkanensis* **sp. nov.** overlaps with the SVL of male *F. sengupti* (23.0-37.8 mm, Meghalaya, India; Purkayastha & Matsui, 2012). However, the new species clearly differs from it in relative finger lengths Ⅱ < Ⅳ < Ⅰ < Ⅲ and shagreened dorsum, vs. Ⅱ < Ⅰ < Ⅳ < Ⅲ and warty dorsum.

The following species have greater male SVL values than that of *F*. *muangkanensis* **sp. nov.**: *F. mysorensis* from India (37.0 mm; Dutta, 1997, as *Limnonectes*), *F. teraiensis* from Nepal (40.1-50.5 mm; Matsui et al., 2007), and *F. murthii* (35.0 mm; Dutta, 1997, as *Limnonectes*) and *F. nilagirica* (34.7-42.2 mm), two species endemic to India.

*F*. *nilagirica*, *F. caperata*, and *F. mudduraja* from the Western Ghats differ from the new species based on their body sizes and proportions and by the presence of warts and dermal ridges on the dorsum (Kuramoto et al., 2007). *F*. *nilagirica* is a large-bodied species and can be easily distinguished from the small-bodied *F.* *muangkanensis* **sp. nov.** (SVL=33.5 mm in males); it can be further distinguished from the new species by numerous warts and dermal ridges on the dorsum (vs. smooth to shagreened dorsum with glandular warts on the posterior part) and by relatively smaller eyes, EHD/SVL=0.1 (vs. EHD/SVL=0.1). *F*. *caperata* is smaller than the new species, with a mean SVL of 29 mm in males and 33 mm in females (vs. SVL 33.5 mm in males and 40.3 mm in females). It can also be differentiated from *F.* *muangkanensis* **sp. nov.** by relative finger lengths (Ⅳ < Ⅱ < Ⅰ < Ⅲ vs. Ⅱ < Ⅳ < Ⅰ < Ⅲ). *F*. *mudduraja* can be distinguished from the new species by its larger female body size with a mean SVL of 45 mm (vs. 40.0-40.9 mm); no information on male SVL exists for *F. mudduraja*. The species also differs from *F.* *muangkanensis* **sp. nov.** by having a head width greater than head length, HW/HL=1.1 (vs. head width less than head length, HW/HL=0.7).

The new species can be easily distinguished from *F. teraiensis* of Nepal and northeast India by its smaller body size (SVL 33.5 mm vs. 37.8-44.1 in males; Howlader, 2011), relative finger lengths (Ⅱ < Ⅳ < Ⅰ < Ⅲ vs. Ⅱ=Ⅳ < Ⅰ < Ⅲ; Howlader, 2011), and having a head width less than head length (HW/HL=0.7 vs. HW/HL=1.0, i.e., head width almost equal to head length).

The two species from Sri Lanka, *F. kirtisinghei* and *F. greenii*, can be easily differentiated from *F*. *muangkanensis* **sp. nov.** by their dorsums being covered with well-developed, long, continuous dermal ridges (vs. shagreened dorsum with rare low dorsal ridges, never forming continuous rows).

Further comparisons of *F*. *muangkanensis* **sp. nov.** with other species in the region is complicated due to their unclear taxonomic status. The locality of *F. brevipalmata*, originally designated as Bago, Myanmar, appears to be uncertain, and might also include the Western Ghats of southern India; thus, the status of this taxon is unclear (AmphibiaWeb, 2017; Boulenger, 1920). Furthermore, *F. sauriceps* and *F. parambikulamana* are endemic to Kerala and Karnataka in southern India. Both are known only from holotypes that appear to have been lost. Regardless, the numerous differences in morphology, coloration, and mtDNA gene sequences support the recognition of the specimens collected from Thong Pha Phum as a new species.

## DISCUSSION

Although our discovery of *F*. *muangkanensis* increases the total number of *Fejervarya* species of Thailand to nine (Suwannapoom et al., 2016), the diversity of this group may still be underestimated. For example, previous molecular studies have identified several distinct lineages diverged from closely related, recognized species, including *Fejervarya* sp. hp3 from Pilok, Thailand, and *F.* sp. hp2 from Bangkok, Thailand (Kotaki et al., 2010). Future studies should examine morphological characteristics of specimens from these regions in detail to confirm their taxonomic identities. Moreover, as many areas of Thailand are still poorly or never surveyed for amphibian diversity, especially in southern Thailand, unrecognized diversity of the genus could still exist. Closer inspections of previously collected congeners from these regions are necessary to better understand amphibian diversity in Thailand, which could help to manage and conserve this unique diversity effectively.

## ACKNOWLEDGEMENTS

We thank the staff at Prof. Ya-Ping Zhang's laboratory (KIZ, CAS) for technical support. The Institute of Animals for Scientific Purpose Development (IAD) issued permission to collect specimens in Thailand (permit number U1-01205-2558).
